# Identifying main effects and epistatic interactions from large-scale SNP data via adaptive group Lasso

**DOI:** 10.1186/1471-2105-11-S1-S18

**Published:** 2010-01-18

**Authors:** Can Yang, Xiang Wan, Qiang Yang, Hong Xue, Weichuan Yu

**Affiliations:** 1 Laboratory for Bioinformatics and Computational Biology, Department of Electronic and Computer Engineering, The Hong Kong University of Science and Technology, Clear Water Bay, Kowloon, Hong Kong, PR China; 2 Department of Computer Science and Engineering, The Hong Kong University of Science and Technology, Clear Water Bay, Kowloon, Hong Kong, PR China; 3 Department of Biochemistry, The Hong Kong University of Science and Technology, Clear Water Bay, Kowloon, Hong Kong, PR China

## Abstract

**Background:**

Single nucleotide polymorphism (SNP) based association studies aim at identifying SNPs associated with phenotypes, for example, complex diseases. The associated SNPs may influence the disease risk individually (main effects) or behave jointly (epistatic interactions). For the analysis of high throughput data, the main difficulty is that the number of SNPs far exceeds the number of samples. This difficulty is amplified when identifying interactions.

**Results:**

In this paper, we propose an Adaptive Group Lasso (AGL) model for large-scale association studies. Our model enables us to analyze SNPs and their interactions simultaneously. We achieve this by introducing a sparsity constraint in our model based on the fact that only a small fraction of SNPs is disease-associated. In order to reduce the number of false positive findings, we develop an adaptive reweighting scheme to enhance sparsity. In addition, our method treats SNPs and their interactions as factors, and identifies them in a grouped manner. Thus, it is flexible to analyze various disease models, especially for interaction detection. However, due to the intensive computation when millions of interaction terms needs to be searched in the model fitting, our method needs to combined with some filtering methods when applied to genome-wide data for detecting interactions.

**Conclusion:**

By using a wide range of simulated datasets and a real dataset from WTCCC, we demonstrate the advantages of our method.

## Background

Rapid Improvements of high-throughput genotyping technologies enable us to detect genetic variations with much finer resolution than before. In genome-wide association (GWA) studies of complex diseases, a few thousands samples are collected and hundreds of thousands of single nucleotide polymorphisms (SNPs) have been genotyped for each sample [1].

Researchers have been investigating disease-associated gene mapping for decades and various approaches have been proposed. However, most of them have used a single-SNP based strategy, in which each SNP is analyzed individually (see [2] for a comprehensive review). Due to the sophisticated regulatory mechanism encoded in the human genome, it is widely agreed that complex traits are typically caused by multiple genetic variations. One type of genetic variation influences the traits individually. This is known as main effects. Another type of genetic variation is that SNPs may show little effect individually, but strong effects jointly. This is known as epistasis or multilocus interactions [3]. Therefore, multi-locus based approaches are believed to have higher power than single-locus based ones. Identifying epistatic interactions arises as an important problem in multi-locus based approaches [4].

Recently, an increasing number of research has reported the presence of epistatic interactions in complex diseases, such as type-2 diabetes [5]. In order to detect epistatic interaction, various computational and statistical methods have developed [4]. For example, Nelson et al. [6] proposed a combinatorial partitioning method (CPM) that enumerated multi-locus genotypes and evaluated them with phenotypes. Culverhouse et al. [7] proposed a restricted partitioning method (RPM) to improve the efficiency of CPM. Millstein et al. [8] developed a testing framework when epistasis is present. Ritchie et al. [9] proposed a multifactor-dimensionality reduction (MDR) method that identified interactions based on classification accuracy through exhaustive search. Zhang and Liu [10] proposed a Bayesian epistasis association mapping (BEAM) method to address the issue of epistasis mapping in genome-wide scale by using Markov Chain Monte Carlo (MCMC) method. In spite of their promising performance, most of these methods only show their successes in association studies on small-scale data sets.

From our view, detecting disease-associated SNPs and their interactions can be cast as the variable selection problem in the framework of regression analysis. Standard tools for variable assessment are the methods of multivariate regression. In traditional applications of multivariate regression, the number of variables is less than the number of samples. In the context of SNP-based disease association studies, however, the number of SNPs is far more than the number of samples, making it difficult or even impossible to directly apply standard multivariate regression methods.

It is widely agreed in GWA studies that only a small fraction of SNPs is disease-associated. In the multivariate regression framework, this implies that most regression coefficients should be zero. This motivates us to impose a sparsity constraint to the regression model. In addition, SNPs are bi-allelic markers (i.e., with allele *A *and *a*). Each SNP has only three genotypes: two homozygous genotypes (*AA *and *aa*) and one heterozygous genotype (*Aa*). Therefore, each SNP can be naturally treated as a three-level factor and be coded with three dummy variables. Similarly, the interaction between two SNPs can be treated as a nine-level factor. In order to encourage sparsity on factors (groups of variables) rather than a single dummy variable, we impose a group constraint on the set of dummy variables that represent a disease model (e.g., a single locus model or a two-locus model). Hence, we propose an Adaptive Group Lasso (AGL) method to identify main effects and epistatic interactions from large-scale SNP data. Since Lasso [11] is well known for imposing a sparsity constraint at the variable level, we employ Group Lasso [12,13] to impose the sparsity constraint at the factor level, and develop adaptive reweighting to enhance the sparsity and to reduce false positive finding.

## Results and discussion

In this section, we evaluate the performance of our method using both simulated and real data. In simulation studies, we compare our method with some recent competitors under a wide range of epistatic models. For the real case-control study, we use the rheumatoid arthritis (RA) data set from the Wellcome Trust Case Control Consortium (WTCCC).

### Simulation studies

In simulation studies, we mainly compare our method AGL with Lasso, BEAM and MDR.

We choose Lasso [14,15] for comparison due to the close relationship between our method and the two Lasso methods. For the identification of main effects, they presume additive, dominant or recessive effects when fitting the Lasso model. For the identification of interactions, Hoggart et. al [14] do not consider this issues and Wu et. al [15] only restrict themselves to the SNPs with strong main effects. Our method is different from their methods in the following sense:

(1) We do not presume particular types of main effects and interactions. Thus, our model is more flexible.

(2) We impose a sparsity constraint at the factor level instead of at the variable level.

(3) Our model includes all possible interactions and is able to identify interactions with weak main effects.

We also compare with BEAM [10] which arises as a powerful epistasis mapping method. Both methods share the concept of three SNP classes: unassociated SNPs, SNPs with main effects and SNPs with interactions. BEAM builds a Bayesian partition model based on these three classes. It is worth mentioning that there is only a single group of interacting SNPs in the BEAM model. To identify multiple interacting groups, BEAM implicitly makes use of MCMC to visit possible interactions. We explicitly allow multiple groups of interacting SNPs and impose additive effects between those groups. Comprehensive comparison studies between BEAM and other related methods have been carried out in [10].

Due to the limited space, the comparison with MDR is given in the supplementary. We further conduct null simulation to estimate the type I error rate of our method.

In the following experiments, we use five-fold cross-validation in model fitting process. We use Bonferroni correction to adjust our p-value and set the significance threshold as 0.3 in simulation studies. In the released version of BEAM, the threshold is set as 0.3. Thus, we choose the same threshold.

#### Comparison with Lasso

We conduct experiments under two scenarios.

• Scenario 1: Identification of main effects.

To illustrate our point, we consider two disease models M1-1 and M1-2, as given in Table 1. M1-1 is a multiplicative model used in both [16] and [10]. M1-2 is proposed in [17] to exhibit the interference effect. We choose these two models with different minor allele frequencies (MAF) to illustrate the influence of model specification when identifying main effects. Under each model setting, we generate 100 data sets which contains 1000 SNPs.

**Table 1:** Two epistasis models (left: M1-1; right: M1-2).

Model 1-1	AA	Aa	aa	Model 1-2	AA	Aa	aa
BB	*α*	*α*	*α*	BB	*α*	*α*	*α*
Bb	*α*	*α*(1 + *θ*)^2^	*α*(1 + *θ*)^3^	Bb	*α*	*α*(1 + *θ*)	*α*
bb	*α*	*α*(1 + *θ*)^3^	*α*(1 + *θ*)^4^	bb	*α*	*α*	*α*(1 + *θ*)

**Figure 1:**
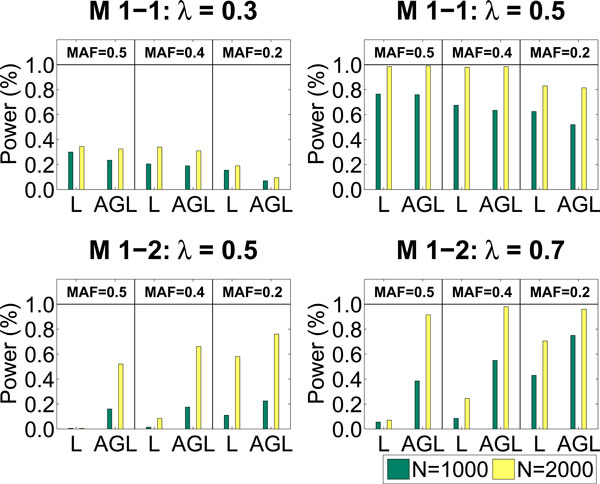
**The performance comparison between Lasso and AGL on identification of main effects**. We generate 100 replicates under each setting. Both 1000 samples and 2000 samples with balanced design are simulated. For Model 1-1, the main effect can be well approximated by the additive effect. Thus, Lasso outperforms AGL slightly. For Model 1-2, the main effect can not be approximated by additive, dominant or recessive effect. Thus, AGL outperforms Lasso significantly.

The performance of Lasso and AGL is summarized in Fig. 1. The power of each method is calculated as the ratio between the number of successful identifications of disease loci and the number of data sets. Lasso performs slightly better than our method for model M1-1. But it performs much worse than our method for model M1-2. Here are the reasons: Firstly, we impose additive effects of SNPs in Lasso model fitting and then perform statistical tests with *df *= 1. Secondly, for AGL we do not assume additive effects of SNPs but use a more general model structure (see our model (2) in Method) and perform statistical tests with *df *= 2. Therefore, Lasso performs better under M1-1 since the imposed additive structure in Lasso agrees well with the structure in model M1-1. M1-2 exhibits interference effect which can not be well approximated by additive, dominant or recessive effect. Lasso performs much worse than Adaptive Group Lasso due to the model mismatch.

• Scenario 2: Identification of interaction effects.

**Figure 2:**
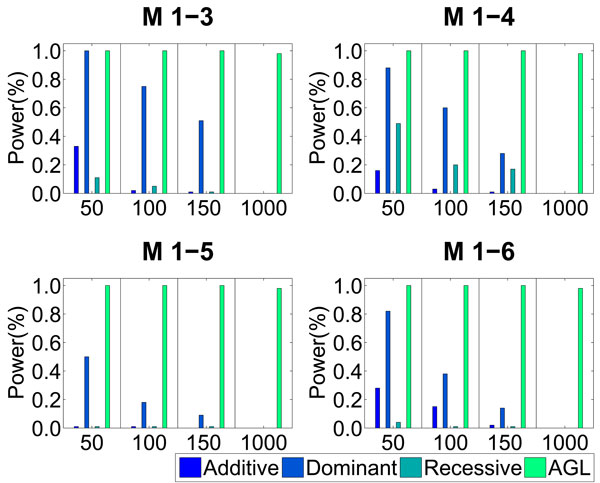
**The performance comparison between Lasso and AGL on pure epistasis model**. Different main effects (additive, dominant and recessive) and their interactions are taken into consideration when fitting the Lasso model. The number in the x-axis is the number of SNPs simulated in the experiments. Lasso with a presumed model performs poorly with increasing number of noise SNPs, while AGL is robust under all settings.

The model mismatch problem of Lasso is more serious when identifying interactions. Here we consider four epistatic models M1-3 ~ M1-6 used in [18], as given in Table 2. Here we report the performance of Lasso and AGL under these four models. The comparison results of other epistatic models in [18] are similar. For Lasso method, we take different main effects and their interactions into consideration during Lasso model fitting. Here we use our interaction model (see model (7) in Method). We simulate two associated SNPs based on the disease models, and gradually increase the number of noise SNPs. Fig. 2 summarizes the experimental results. The power is calculated as the proportion of the 100 data sets in which interactions of the disease associated SNPs are detected. Lasso with presumed main effects (additive, dominant or recessive) loses its power rapidly as the number of noise SNPs increases, while AGL keeps its power when more noise SNPs are involved in model fitting.

**Table 2:** Four pure epistatisis models used in [18].

Model 1-3	*h*^2^**=** 0.3, *p*_*a *_= 0.4**, ***q*_*b *_= 0.4			Model 1-4	*h*^2^**= **0.2, *p*_*a*_= 0.4, *q*_*b*_****= 0.4		
			
	AA	Aa	aa		AA	Aa	aa
BB	0.077	0.689	0.417	BB	0.086	0.536	0.641
Bb	0.763	0.150	0.491	Bb	0.677	0.275	0.096
bb	0.196	0.657	0.247	bb	0.219	0.413	0.712
Model 1-5	*h*^2 ^= 0.1, *p*_*a *_= 0.4, *q*_*b *_= 0.4			Model 1-6	*h*^2 ^= 0.05, *p*_*a *_= 0.4, *q*_*b *_= 0.4		
			
	AA	Aa	aa		AA	Aa	aa
BB	0.068	0.299	0.017	BB	0.005	0.179	0.251
Bb	0.289	0.044	0.285	Bb	0.211	0.100	0.026
bb	0.048	0.262	0.174	bb	0.156	0.098	0.156

Here we only provide four pure epistatisis models used in comparison with Lasso. The complete model list used in comparison with BEAM is provided in our supplementary document (please see Additional File 1).

Generally speaking, if the underlying interaction could be well characterized by Lasso with a presumed model structure, e.g., additive model, then the statistical power of Lasso would be higher than that of AGL because Lasso uses less degree of freedom. However, since the underlying interaction is generally unknown and its possible pattern may cover a wide range of spectrum [19], AGL can serves as a valuable tool for discovering interactions in larger model space. Hence, AGL and Lasso may be complementary to each other in GWA studies.

**Figure 3:**
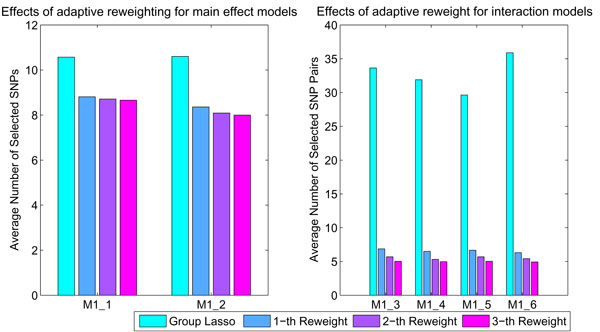
**The reweighting effect of Adaptive Group Lasso**. The reweighting greatly reduces the number of selected SNP pairs after the first iteration. This effect is more obvious when identifying interactions in M1-3, M1-4, M1-5, M1-6.

We show the effect of adaptive reweighting in Fig. 3. The first reweighting greatly reduces the number of selected dummy variable groups and the reweighting process converges in a few iterations (typically less than 5 iterations). The adaptive reweighting process reduces the number of unassociated groups and leads to more accurate p-value calculation in the statistical testing.

On the other hand, however, it can also be seen that unassociated groups may enter the final model even after adaptive reweighting. Hence, the selected groups in the final model may not be associated with the phenotype. In this regard, the significance assessment is critically needed.

#### Comparison with BEAM

We shall not compare our method with BEAM when genetic heterogeneities are present since BEAM is not developed to handle these cases (the authors of BEAM had made it clear in [10]). We shall compare with BEAM from two perspectives:

1. The ability of detecting epistatic interactions when the main effect is weak or even absent.

2. The ability of detecting multiple interactions.

#### Detecting epistatic interactions with weak main effect

A wide range of interaction models without marginal effects has been discussed in [19]. Here we consider the 40 pure epistatic models in [18] to compare the performance between AGL and BEAM. The details of these models are available in the supplementary document. The heritability *h*^2^(see definition in [19]) of these 40 models ranges from 0.05 to 0.2 and the

MAF ranges from 0.2 to 0.4. We use 100 data sets for each disease model. There are 200 cases, 200 controls, and 1000 SNPs in each data set.

**Figure 4:**
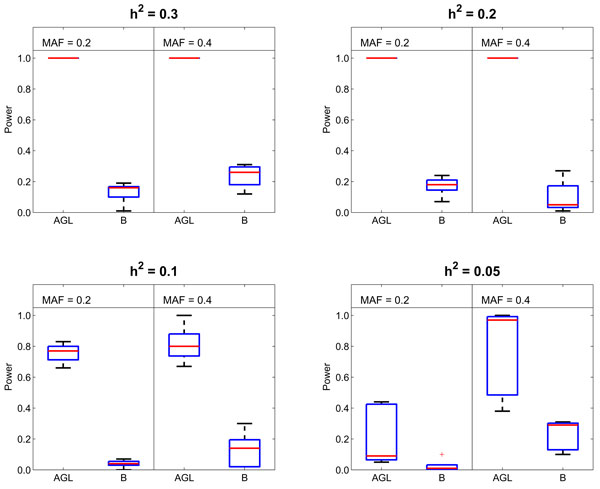
**The performance comparison between AGL and BEAM (B) based on pure epistatic models**. We generate 100 datasets for each model. Each dataset contains 400 sample (*N*_*u *_= 200, *N*_*d *_= 200) and 1000 SNPs. BEAM runs 5 × 10^6 ^Markov Chain Monte Carlo iterations which are 10 times of pairwise exhaustive search. The comparison shows that our method outperforms BEAM for these pure epistatic models.

The comparison between our method and BEAM in Fig. 4 shows that AGL is superior to BEAM for detecting epistatic interactions without main effects. For the models with *MAF *= 0.2, 0.4 and *h*^2 ^≥ 0.1, the power of our method is above 95%, while that of BEAM is roughly 20%. The performances of the two methods degrade as the heritability *h*^2 ^decreases: the power of BEAM is lower than 5% for the models with *MAF *= 0.2 and *h*^2 ^≤ 0.1, while the power of our method still remains at about 75% for some of these models and is even higher for the models with *MAF *= 0.4.

Our model includes all possible interactions (1000 × 999/2 interactions) in the model fitting process, so there is no chance to miss interesting interactions. The good performance of our model is due to the group-sparsity constraint: It identifies interactions in a grouped manner. This is very helpful to weaken the influence of noise SNPs.

The poor performance of BEAM is not due to the statistical testing power of the B-statistics [10], but the sampling efficiency. We carefully examined the interactions in the disease models with *h*^2 ^≥ 0.1: Those interactions are very significant even after the Bonferroni correction of B-statistics. Notice that BEAM runs 5 × 10^6 ^MCMC iterations which are 10 times of pairwise exhaustive search in the simulation study. Thus, our conjecture is that MCMC might converge too slowly to find the ground truth (We provide some evidence in the supplementary to support our conjecture).

#### Detecting multiple interactions

Another disadvantage of BEAM is that it only allows a single interacting group in its Bayesian partition model. To compensate for this limitation, BEAM uses MCMC sampling strategy to visit possible interactions during model optimization. In contrast, our approach allows multiple interacting groups and imposes additive effects for these interactions. This flexibility enables us to have a higher power to identify multiple interactions. Due to the limited space, we show our comparison result of detecting multiple interactions in the supplementary.

#### Null simulation study

To validate the use of our p-value and to estimate the type-I error, we conduct null simulation studies in two cases:

• Case 1: We generate 100 null datasets. Each dataset contains 10 K SNPs and 1000 samples. All the SNPs are generated independently with MAF uniformly distributed in [0.05, 0.5]. In this case, the nominal type-I error rates should be 10, 20, 30 per one million SNPs for significance thresholds at 0.1, 0.2, 0.3.

• Case 2: We use genomeSIMLA [20] to simulate the SNP data based on the marker information on the Affymetrix 500 K chip from human chromosome 1. Linkage disequilibrium (LD) exists among SNPs. We also generate 100 null datasets, each of which contains 38836 SNPs and 1000 samples. Due to LD pattern, the error rate should lower than the nominal error rate.

We summarize the type-I error of our model (2) in Table 3. For Case 1, Our results are reasonable for the three nominal levels. For Case 2, our type-I error rates show that our method is conservative when LD exists.

**Table 3:** Null simulation: empirical type-I error from 100 simulated data sets.

**Cases**	**Error rates per one million SNPs of different thresholds after Bonferroni correction**		
	
	***threshold *= 0.1**	***threshold *= 0.2**	***threshold *= 0.3**
Case 1	11	18	28
Case 2	3.08	4.89	7.47

The nominal type-I error rates should be 10, 20, and 30 per one million SNPs for significance thresholds at 0.1, 0.2, and 0.3, respectively.

### Analysis of WTCCC data

#### Main effects

Current version of BEAM software can not handle WTCCC data on the genome-wide scale. To compare with BEAM on the real data, we apply our main effect model (2) and BEAM to analyze WTCCC Rheumatoid Arthritis (RA) data in the chromosome-wise manner. For BEAM, we run 10^8 ^MCMC and set the significant threshold as 0.3 after Bonferroni correction. BEAM does not report any interactions. The identified main effects of the two methods agree with each other. Some of them are given in Table 4 and the details are given in the supplementary document. The significant result from WTCCC [1] could be reproduced.

**Table 4:** Some significant SNPs identified by our method on WTCCC RA data. These SNPs covers the regions which are moderate associated with RA as reported in [1].

SNPs	Location	Related Genes	P-value
rs7539166	1p36	TMEM51	< 10^-30^
rs384843	1p36	NBPF3	5.2 × 10^-9^
rs7516721	1p36	GPR3	7.7 × 10^-14^
rs4959053	6p21	PSORS1C1	< 10^-30^
rs6457617	6p21	MHC region	1.3 × 10^-15^
rs6973565	7q32	PLXNA4	1.2 × 10^-9^
rs10250029	7q32	CHCHD3	< 10^-30^
rs10751815	10p15	ADARB2	< 10^-30^
rs4266996	10p15	unknown	< 10^-30^
rs17147777	10p15	unknown	< 10^-30^
rs8129909	21q22	IGSF5	< 10^-30^
rs16999716	21q22	DSCAM	9.2 × 10^-9^
rs4542939	21q22	DSCAM	8.3 × 10^-9^
rs13047947	21q22	PDE9A	< 10^-30^
rs140344	22q12	unkown	< 10^-30^
rs5749509	22q12	SYN3	< 10^-30^
rs6518796	22q12	SYN3	< 10^-30^

• WTCCC reports that SNP rs6457617 located at 6p21 shows a very strong association. Our experiment verifies this result.

• We do not identify SNP rs6679677 located at 1p13 reported by WTCCC. Instead, we identify two SNPs rs7551793 and rs948620 near SNP rs6679677. The signals of these two SNPs are much stronger than rs6679677.

• We also identify SNPs in the moderate association regions reported in [1]. We summarize the result in Table 4.

#### Interactions

Detecting interactions in genome-wide scale is very challenging and multi-stage strategies are often explored. For example, MDR [9] usually is combined with TuRF [21] which serves as a filter to remove those noise SNPs. Currently, our method AGL can not be directly applied to genome-wide scale SNP data since it is too computationally intensive to exhaustively search for all SNP pairs. As suggested in simulation study, our method keeps its statistical power when about 500,000 SNP pairs are considered in our model. Thus, the main difficulty is the computation burden of searching for all SNP pairs. Thus, a filtering method is necessary for our method.

For identification of epistatic interactions, we focus on two candidate regions: 6p21 and 7p21. These two regions are reported in our previous work named SNPHarvester [22] which is a filtering method. Here we apply our interaction model (7) and report some SNP interactions in Table 5.

**Table 5:** Some significant SNP groups identified by our method in the candidate regions on WTCCC RA data.

SNP Groups	Location	Related Genes	P-value
(rs4988822, rs3135392)	6p21.3	(HLA-DRA, HLA-DRA)	3.33 × 10^-15^
(rs17429127, rs2157082)	6p21.3	(HLA-DRA, HLA-DRA)	< 10^-30^
(rs1358169, rs6460831)	7p21.3	(THSD7A, THSD7A)	< 10^-30^

• For the region 6p21, we select a segment covering the SNPs reported in [22]. This segment contains 250 SNPs from rs3135366 to rs461338. We enumerate all possible interactions and include them in our model. Our method reports two interacting pairs: (rs4988822, rs3135392) and (rs17429127, rs2157082). These SNPs are related to gene HLA-DRA. The result in [23] reports that there is a strong association between RA and HLA gene family. Notice that the two SNPs rs4988822 and rs3135392 cannot be identified by univariate analysis due to their weak main effects (Their p-value is at the level of 10^-3 ^given by univariate analysis). However, they do show a strong interaction. We also run BEAM on the selected segment (5 × 10^6 ^MCMC). BEAM reports (rs9469220, rs3957146) and (rs3129872, rs9272723) as the two most significant interactions based on the B-statistics. We carefully check these two pairs based on the logistic regression model. We find that the interaction of the SNP pair (rs9469220, rs3957146) is very weak using the standard *χ*^2 ^test based on logistic regression models with *df *= 4, while the interaction of the SNP pair (rs3129872, rs9272723) is strong. We further explore the reason why our method does not report the interacting pair (rs3129872, rs9272723). We observe that SNP rs9268645, which is highly correlated (strong LD) with SNP rs3129872, enters our model as a main effect term. Consequently, the pair (rs3129872, rs9272723) does not enter the model as a interaction term. This shows that the SNP pair (rs3129872, rs9272723) should not be reported as an interacting pair.

• For the region 7p21, we select a segment which covers the SNPs reported in [22]. The segment contains 250 SNPs from SNP rs1076224 to SNP rs1548882. We analyze this region and report one interacting pairs (rs1358169, rs6460831). The two SNPs rs1358169 and rs2526100 are related to gene THSD7A on chromosome 7, which has been reported to be associated with bone mineral density [24]. This shows plausible biological relevance. We also run BEAM on the segment. But it does not report any interaction.

• Neither BEAM nor our method finds significant interactions in the region 6q23.

The definition of interactions is not consistent in literatures. For example, the interaction effect of two SNPs is define via logistic regression models of genotypes and their combinations [3], while it is also defined via models of haplotypes [25]. The interactions reported above are based on the definition of interaction of a SNP pair in [3]. We extend this definition such that we can simultaneously handle interactions of multiple SNP pairs. The details are given in the Method section. We realize that the interacting SNPs reported above are close to each other on the physical map. This type of interaction effects may be caused by the haplotype effect. Detecting interactions of genes in different genome regions by analyzing genome-wide SNP data is still under investigation [4].

## Conclusion

In this paper, we proposed an Adaptive Group Lasso method for large-scale SNP data analysis. The novelty of our method is that it analyzes SNPs and their interactions simultaneously. It imposes a sparsity constraint at the group level and enables us to identify associated SNPs (especially for interacting SNPs) from large-scale SNP data robustly. We show that our method outperforms its recent competitors in both simulation studies and real application.

The limitation of our method is that the interaction model can not be directly applied to genome-wide scale SNP data analysis. The main difficulty comes from the computation burden of searching for all SNP pairs. There are two possible solutions to solve this issue. One solution is to make use of some filtering method to reduce the number of SNPs to a manageable size, for example, [22]. Another solution is incorporating biological information. Pathway information [26] provides a biological clue to narrow down the search range for interaction detection. We shall investigate this in our future work.

## Methods

SNPs are high-density bi-allelic markers. We use capital letters (e.g., *A *and *B*) and lowercase letters (e.g., *a *and *b*) to denote major and minor alleles, respectively. We also use *G*_1 _to denote the collection of all three genotypes of one SNP and use *G*_2 _to denote the collection of all nine combinations of two SNPs:(1)

Our target is to identify disease-associated SNPs and their interactions. Researchers may have different understandings of epistatic interaction. To be clear, Our definition of interaction refers to the deviation from the additive model of multiple SNPs. In other words, the interaction effects refers to the phenotype variation that can be explained by joint effects of multiple SNPs but not by their main effects. This is consistent with the definition given in [3]. We achieve our goal through addressing the following key issues:

• **Model: **How to model the relationship between genotype and phenotype?

• **Optimization: **How to optimize the model structure?

• **Significance assessment: **How to test the significance of the detected association?

In the following, we first present our model for identification of main effects and epistatic interactions. Then, we give an optimization algorithm for large-scale SNP data analysis. Finally, we describe a method to assess the significance of the SNPs identified by our model.

### The adaptive group Lasso model for identification of main effects

Suppose *N *samples with *N*_*d *_cases and *N*_*u *_controls have been genotyped at *L *loci for an association study. Now we have a design matrix **X **collecting these *N *samples and a response variable **y **∈ ℝ^*N *^indicating a sample from case or control. Since A SNP has only three genotypes, we treat it as a factor and code it with three dummy variables. We use [1 0 0] to code genotype *AA*, [0 1 0] to code genotype *Aa *and [0 0 1] to code genotype *aa*. Then the design matrix **X **becomes a *N *× (*L *× 3) matrix. We shall use **X**_*j*_, a submatrix of size *N *× 3, to denote the columns of **X **corresponding to the *j*-th SNP. Similarly, **X**_*ij*_, a submatrix of size 1 × 3, corresponds to the *i*-th sample and the *j*-th SNP. We propose an Adaptive Group Lasso logistic regression model (AGL) for main effect identification:(2)

where *γ *is a real value parameter controlling the trade-off between the likelihood and the constraint, *p*_*j *_= 3 is the number of dummy variables used to coding the *j*-th SNP for *j *= 1, ⋯, *L*, ***β***_*j *_= [*β*_*j*, *AA*_, *β*_*j*, *Aa*_, *β*_*j*, *aa*_], , and l(***β***) is the log-likelihood of logistic regression:(3)

For convenience, we define an *active set * = {*j*|***β***_*j *_≠ 0}. We use an iterative algorithm to adaptively assign weights *w*_*j *_in (2) as given in Algorithm 1.

From the iterative algorithm, the penalty weight is adjusted according to its previous estimation. If the current estimation  is small, i.e., *SNP*_*j *_is less likely to be associated with disease, then the penalty weight should increase to prevent *SNP*_*j *_from entering the model in the next iteration, and vice versa. The theoretical justification is given in the supplementary document.

**Algorithm 1 **Adaptive Reweighing Algorithm of Group Lasso

1. Set the iteration count *m *= 0. Initially set  = 1, *j *= 1, ⋯, *L*.

2. Solve problem (2) to obtain *β*() and the active set , where  is determined by cross-validation.

3. (1) Update the weight:(4)

(2) Remove *SNP*_*j*_, when *j *∉ .

4. If the active set  does not change, stop; otherwise increment *m *and go to step 2.

The proposed model has following characteristics:

• Flexibility: Due to the dummy variable representation, our model is flexible to analyze different main effects (additive, dominant, recessive, interference [17]) in a unified way without presuming one particular type of effect. This flexibility will be pronounced when identifying epistatic interactions.

• Sparsity: The sparsity constraint  comes from the fact that most SNPs are unassociated. Notice that  imposes a constraint to select a group of dummy variables rather than a single one. The weight *w*_*j *_is assigned adaptively in Algorithm 1 to enhances the sparsity at the factor level. If without reweighting, too many noise SNPs would enter the model in GWA studies.

### The adaptive group Lasso model for identification of epistatic interactions

#### Definition of interactions

Interactions between *SNP*_1 _and *SNP*_2 _are often defined via logistic regression models as in [3]. Let  and  be the dummy variable coding for genotype *G *of *SNP*_1 _and *SNP*_2_, respectively. The main effect logistic regression model of *SNP*_1 _and *SNP*_2 _is:(5)

The full logistic regression model of *SNP*_1 _and *SNP*_2 _is:(6)

Let *L*_*M *_and *L*_*F *_be the log likelihood of the main effect model and the full model, respectively. According to the likelihood ratio test, interaction effects are defined via the difference of the log likelihood of these two models, i.e., *L*_*F *_- *L*_*M*_. Hence, interaction effects can be interpreted as departure from linear models naturally [4].

#### Modelling interactions

The model for identification of epistatic interactions is an extension of model (2): Interaction terms are further included in a grouped manner. We treat the combination of two SNPs as a 9-level factor and use 9 dummy variables to code them. Let *J*_1 _be the index set of all SNPs:

*J*_1 _≜ {1, 2,⋯, *L*}, and *J*_2 _be the index set of all pairwise interactions of *L *SNPs:

*J*_1 _≜ {(1, 2), (1, 3)⋯, (*L *- 1, *L*)}. The design matrix **X **becomes a *N *× (*L *× 3 + *L *× (*L *- 1)/2) matrix, i.e., , where  and  are the sub design matrices collecting main effect groups and interaction groups, respectively. Similarly, We use  with *j*_1 _∈ *J*_1 _to denote the *i*-th sample and the *j*_1_-th group in  and  with *j*_2 _∈ *J*_2 _to denote the *j*_2_-th group in . We propose the following model to identify epistatic interactions:(7)

where  for *j*_1 _∈ *J*_1_,  for *j*_2 _∈ *J*_2_,  (definitions of *G*_1 _and *G*_2 _are given in Eqs. (1)), and ℓ(***β***) is the log-likelihood of logistic regression:(8)

The proposed model structure has the following characteristics:

• Our interaction model integrates the analysis of SNPs with main effects and interacting SNPs, and unassociated SNPs are prevented from entering the model by the sparsity constraint. Recall that interaction refers to the phenotype variation that can be explained by joint effects of multiple SNPs but not by their main effects, model (7) includes main effects to discourage the interaction terms to enter the model, when their effects can be mostly explained by the main effects. However, this may not be able to completely prevent spurious interactions from entering the model. The reason is that the interaction terms may explain more variances than their corresponding main effects. Hence, they are more likely to enter the final model even when they are penalized more heavily ( for *j*_1 _∈ *J*_1_,  for *j*_2 _∈ *J*_2_). To overcome this difficulty, we resort to the statistical test of interaction effects.

• By using dummy variables, our model is flexible to model various interactions, including all epistatic models described in [17]. Both main effect terms and interaction terms enter the model in a grouped manner. Thus, our model is insensitive to the noise occurring at one level of the genotype combinations *G*_1 _and *G*_2_.

• Our model imposes additive effects for these interactions. Simultaneous analysis of all interactions achieves under this model structure.

### Optimization algorithm

In our Adaptive Reweighing Algorithm of Group Lasso (Algorithm 1), we need an algorithm to solve optimization problem (2) efficiently. We make use of the coordinate descent algorithm [13,27]. The advantage of the coordinate descent algorithm is that it has a closed-form solution of the least square problem when updating one group at a time. Therefore, it is suitable for large-scale data analysis. For the log-likelihood of logistic regression, the iteratively reweighed least square algorithm (IRLS) is efficient: A quadratic approximation is formed to the log-likelihood based on current estimation and the least square problem is solved by the coordinate descent algorithm. This process is repeated until its convergence which is guaranteed [13]. The detail of the algorithm is given in the supplementary.

### Statistical testing

The statistical testing is to determine whether the identified SNPs (i.e., the SNPs in the active set) are significantly associated with the disease

#### Significance tests of main effects

Let  be the SNP set identified under model (2) and *s*_1 _denote the number of identified SNPs in . We use the following strategy to assess the significance of identified SNPs:

1. Re-fit the logistic regression model for the identified SNPs in  and obtain the log-likelihood ℓ_*full*_.

2. Leaving *SNP*_*j *_∈  (*j *= 1, ..., *s*_1_) out, fit the logistic regression models and obtain the log-likelihood ℓ_\*j*_.

3. Obtain p-value of *SNP*_*j *_using *χ*^2 ^test based on the value 2(ℓ_*full *_- ℓ_\*j*_) with the degree of freedom (df) discussed below.

There are several key issues in the above procedure:

1. Since we use three dummy variables to code each SNP, collinearity exists when fitting logistic regression models. To overcome this difficulty, we fit the logistic regression model with a *L*_2 _regularization term(9)

where *γ *is a small number to avoid singularity. Here we set *γ *= 10^-4^.

2. For model (9), the effective degree of freedom is given by(10)

where Γ is a (*s*_1 _+ 1) × (*s*_1 _+ 1) diagonal matrix with diagonal elements [0, *γ*, ..., *γ*], **U **is a diagonal matrix with diagonal elements *p*_*i*_(1 - *p*_*i*_). Here  is evaluated after convergence of the logistic regression model (9) fitting.

3. The degree of freedom of the *χ*^2 ^test for *SNP*_*j *_is , where *df*_*full *_and *df*_\*j *_are the effective degrees of freedom of the logistic regression models with all *s*_1 _SNPs and without *SNP*_*j*_, respectively.

It is worth mentioning that our p-value is obtained after the selecting process in AGL fitting. The selecting process may affect the precision of p-value estimation. We justify our p-value by conducting null simulation later.

#### Significance tests of epistatic interactions

Our Tests of interactions is built upon the definition of interactions. The key point is that the main effects of the two SNPs should not be taken into account when testing their interaction effect. We conduct significance tests of epistatic interactions in the following way:

Let  be the set of the groups identified under model (7) and *s*_2 _is the number of the groups in .

1. Re-fit the logistic regression model for the identified groups in  and obtain the log-likelihood ℓ_*full*_.

2. For each interaction term in  with index *l *(*l *= 1, ..., *s*_2_), fit the logistic regression model with the main effect term replacing the interaction term and obtain the log-likelihood ℓ_\*l*_.

3. Obtain p-value of interaction term *l *using *χ*^2 ^tests based on the value 2(ℓ_*full*_-ℓ_\*l*_) with *df *= *df*_*full *_- *df*_\*l*_.

## Competing interests

The authors declare that they have no competing interests.

## Authors' contributions

CY performed the implementations and drafted the manuscript. XW participated in the experimental design. QY, HX and WY finalized the manuscript. All authors read and approved the final manuscript.

## Supplementary Material

Additional file 1 The supplementary document provide some backgrounds of our method. It also provides details of our optimization algorithm, and comprehensive experimental results. Click here for file
