# Supplementary material for: Identifying main effects and epistatic interactions from large-scale SNP data via adaptive group Lasso
Source: BMC Bioinformatics. 2010 Jan 18;11(Suppl 1):S18. doi: 10.1186/1471-2105-11-S1-S18 (PMC3203332; doi:10.1186/1471-2105-11-S1-S18)
Supplement: Additional file 1 — The supplementary document provide some backgrounds of our method. It also provides details of our optimization algorithm, and comprehensive experimental results. [file 1471-2105-11-S1-S18-S1.pdf]

Supplementary of Identifying Main Effects and Epistatic Interactions From Large-scale SNP Data via Adaptive Group Lasso

Can Yang, Xiang Wan
Qiang Yang, Hong Xue and Weichuan Yu.

October 9, 2009

We give a brief introduction to Lasso-type methods in Section 1. We provide the theoretical justification of Adaptive Group Lasso (AGL) in Section 2. Then we provide details of our optimization algorithm for AGL model fitting in Section 3. We also give more experimental results in Section 4. Finally, we provide the models and their parameter settings used in simulation studies in Section 5.

1 Lasso-type methods

In this section, we shall give a brief introduction to Lasso-type methods.

In the usual linear regression setup, we have a continuous response $\mathbf{y} \in \mathbb{R}^N$, an $N \times p$ design matrix $\mathbf{X}$ and a parameter vector $\boldsymbol{\beta} \in \mathbb{R}^{p+1}$. The Lasso estimator [7] is defined as:

$$\hat{\boldsymbol{\beta}}^L(\gamma) = \arg\min_{\boldsymbol{\beta}} R^{(L)}(\boldsymbol{\beta}) = \underset{\boldsymbol{\beta}=[\beta_0,\beta_1,\cdots,\beta_p]^T}{\arg\min} \left(\frac{1}{2N} \left\| \mathbf{y} - (\beta_0 + \sum_{j=1}^{p} \mathbf{x}_j \beta_j) \right\|_2^2 + \gamma \sum_{j=1}^{p} |\beta_j| \right), \quad (1)$$

where γ is a regularization parameter, $\mathbf{x}_j$ is an $N \times 1$ vector corresponding to the j-th column of the design matrix $\mathbf{X}$ and $\|\mathbf{v}\|_2^2 = \sum_{i=1}^n v_i^2$ for any vector $\mathbf{v} \in \mathbb{R}^n$. The sparsity penalty $\sum_{j=1}^p |\beta_j|$ encourages many β_js to be zero.

When categorical predictors (i.e., factors represented by dummy variables) are present in linear regression, the Lasso solution is not satisfactory since it only selects dummy variables individually. To address this issue, Yuan and Lin [10] proposed to treat the dummy variables as a group, and then imposed a sparsity constraint at the group level.

For convenience, we shall use the following notations to describe the model with grouped variables: Suppose we have a response variable $\mathbf{y} \in \mathbb{R}^N$ and an $N \times p$ design matrix $\mathbf{X}$ collecting N samples with p variables. These p variables are partitioned into J disjoint groups with the

j-th group consisting of p_j variables. Clearly, we have $\sum_j p_j = p$. We shall use $\mathbf{X}_j$, a submatrix of size $N \times p_j$, to denote the columns of X corresponding to the j-th group. Similarly, $\mathbf{X}_{ij}$, a submatrix of size $1 \times p_j$, corresponds to the i-th sample and the j-th group. We also use $\boldsymbol{\beta}_j$ to denote the coefficient vector corresponding to the j-th group.

For the regression problem of J groups with j-th group consisting of p_j variables, the Group Lasso estimator [10] is defined as:

$$\hat{\boldsymbol{\beta}}^{GL}(\gamma) = \arg\min_{\boldsymbol{\beta}} R^{(GL)}(\boldsymbol{\beta}) = \mathop{\arg\min}_{\boldsymbol{\beta}=[\beta_0,\boldsymbol{\beta}_1,\cdots,\boldsymbol{\beta}_J]^T} \left(\frac{1}{2N} \left\| \mathbf{y} - (\beta_0 + \sum_{j=1}^{J} \mathbf{X}_j \boldsymbol{\beta}_j) \right\|_2^2 + \gamma \sum_{j=1}^{J} \sqrt{p_j} \|\boldsymbol{\beta}_j\|_2 \right), \tag{2}$$

where $\boldsymbol{\beta}_j = [\beta_{j,1}, \beta_{j,2}, \cdots, \beta_{j,p_j}]^T$ for $j = 1, \cdots, J$, and β_0 is the intercept in the linear model. Term $\|\boldsymbol{\beta}_j\|_2 = \sqrt{\beta_{j,1}^2 + \beta_{j,2}^2 + \cdots + \beta_{j,p_j}^2}$ imposes a constraint to select a group of variables rather than a single variable. Notice that regularization term $\sum_{j=1}^{J} \sqrt{p_j} \|\boldsymbol{\beta}_j\|_2$ involves the 2-norm $\|\boldsymbol{\beta}_j\|_2$ of $\boldsymbol{\beta}_j$ rather than the squared 2-norm $\|\boldsymbol{\beta}_j\|_2^2$. The "Lasso" term in the name "Group Lasso" refers the sum of the absolute value of the 2-norm, i.e.,

$$\sum_{j=1}^{J} \sqrt{p_j} \left| \|\boldsymbol{\beta}_j\|_2 \right| = \sum_{j=1}^{J} \sqrt{p_j} \|\boldsymbol{\beta}_j\|_2. \tag{3}$$

In the GL model, the group structure is assumed to be known. This structure is explicitly made use of in regularization.

Elastic net introduced in [12] is another type of regularization method to model grouping effects. The Elastic net estimator is defined as:

$$\begin{aligned} \hat{\boldsymbol{\beta}}^{EN}(\gamma_1, \gamma_2) &= \arg\min_{\boldsymbol{\beta}} R^{(EN)}(\boldsymbol{\beta}) \\ &= \mathop{\arg\min}_{\boldsymbol{\beta}=[\beta_0,\beta_1,\cdots,\beta_p]^T} \left(\frac{1}{2N} \left\| \mathbf{y} - (\beta_0 + \sum_{j=1}^{p} \mathbf{x}_j \beta_j) \right\|_2^2 + \gamma_1 \sum_{j=1}^{p} |\beta_j| + \frac{\gamma_2}{2} \sum_{j=1}^{p} \beta_j^2 \right), \end{aligned} \tag{4}$$

Notice that the regularization term $\sum_{j=1}^{p} \beta_j^2$ is the squared 2-norm $||\boldsymbol{\beta}||_2^2$ of $\boldsymbol{\beta}$. In Elastic model (4), no group structure is explicitly imposed. For two variables $\mathbf{x}_i$ and $\mathbf{x}_j$ with correlation ρ, however, their estimated coefficients β_i and β_j will tend to be shrunken to the same value as γ_2 and ρ increase. This is known as the grouping effects encouraged in Elastic net.

In this paper, we are aiming at identify main effects and interactions by analyzing SNP data. For main effects, Lasso and Elastic net can be used with a presumed model structure, e.g., an additive model. Group Lasso can be used without a presumed model structure. This issue has been carefully discussed in the main text of this paper.

For interactions, two-locus models consider 9 genotypes (5) for any two SNPs:

$$G_2 \triangleq \{AABB, AABb, AAbb, AaBB, AaBb, Aabb, aaBB, aaBb, aabb\}. \tag{5}$$

Thus, a two-locus model can be represented by using 9 dummy variables coding 9 genotypes. Further the group structure of these 9 dummy variables arises naturally based on the two-locus model. Hence, Group Lasso model could serve as a basic model for identifying interactions. Based on this consideration, we propose our Adaptive Group Lasso model. Lasso and Elastic net could also be applied for identifying interactions. One way is imposing model structure (e.g.,an additive model) which is the same as what has done for identifying main effects. Another way is using 9 dummy variables coding a two-locus model. Notice that no correlation exists among the 9 dummy variables, i.e., $\rho = 0$. Thus, these dummy variables can not be grouped by Elastic net. In this sense, both Lasso and Elastic net could only do variable selection at the variable level rather than the group level.

2 Theoretical Justification of Adaptive Group Lasso

2.1 Connection with the Majorization-Minimization algorithm

Our iteratively reweighted algorithm is a special case of Majorization-Minimization (MM) algorithms [4]. To establish the connection, consider the problem

$$\min_{\boldsymbol{\beta}} \left(-\ell(\boldsymbol{\beta}) + \gamma \sum_{j=1}^{J} \sqrt{p_j} \log \left(\|\boldsymbol{\beta}_j\|_2 \right) \right), \tag{6}$$

where $\ell(\boldsymbol{\beta})$ is the log-likelihood of logistic regression:

$$\ell(\boldsymbol{\beta}) = \frac{1}{N} \sum_{i=1}^{N} \left[y_i(\beta_0 + \sum_{j=1}^{J} \mathbf{X}_{ij}\boldsymbol{\beta}_j) - \log \left(1 + \exp(\beta_0 + \sum_{j=1}^{J} \mathbf{X}_{ij}\boldsymbol{\beta}_j) \right) \right]. \tag{7}$$

The problem (6) can be rewritten as

$$\begin{aligned} \min_{\boldsymbol{\beta}, \mathbf{v}} \quad & \sum_{j=1}^{J} \sqrt{p_j} \log \left(v_j \right) \\ \text{subject to} \quad & -\ell(\boldsymbol{\beta}) \leq \alpha, \\ & \|\boldsymbol{\beta}_j\|_2 \leq v_j. \end{aligned} \tag{8}$$

The problem is in the form

$$\begin{aligned} \min_{\mathbf{v}} \quad & f(\mathbf{v}) \\ \text{subject to} \quad & \mathbf{v} \in \text{convex set}. \end{aligned} \tag{9}$$

This is a problem that minimizes a concave function on a convex set. Instead of minimizing a concave function, we can minimize its tangent at a local point $\mathbf{v}_0$ since concave functions are upper bounded by its tangent:

$$\min_{\mathbf{v}} f(\mathbf{v}) = \min f(\mathbf{v}_0) + \nabla f(\mathbf{v}_0)(\mathbf{v} - \mathbf{v}_0). \tag{10}$$

Therefore, the problem (8) can be further written as

$$\begin{aligned} \min_{\boldsymbol{\beta},\mathbf{v}} \quad & \sum_{j=1}^{J} \frac{\sqrt{p_j}}{v_j^{(0)}} v_j \\ \text{subject to} \quad & -\ell(\boldsymbol{\beta}) \le \alpha, \\ & \|\boldsymbol{\beta}_j\|_2 \le v_j. \end{aligned} \tag{11}$$

Finally, the problem (11) can be rewritten as

$$\min_{\boldsymbol{\beta}} \left(-\ell(\boldsymbol{\beta}) + \gamma \sum_{j=1}^{J} (w_j \sqrt{p_j} \|\boldsymbol{\beta}_j\|_2) \right), \tag{12}$$

where $w_j = \frac{1}{v_j^{(0)}}$. This is the form we propose in Algorithm 1 in the paper.

2.2 Properties

The theoretical reason for minimizing the negative log-likelihood with a concave penalty (e.g., with log function as in (6)) is given in [1]. The basic idea is that the estimation of important variables should be shrunken less than those of unimportant ones. Based on this idea, Zou proposed the Adaptive Lasso [11] and one-step estimator [13].

Wang et al. [9] have considered the Adaptive Group Lasso in the following form:

$$\min_{\boldsymbol{\beta}} \left(-\ell(\boldsymbol{\beta}) + \sum_{j=1}^{L} \gamma_j \|\boldsymbol{\beta}_j\|_2) \right). \tag{13}$$

They show that the Adaptive Group Lasso could do group selection consistently under some mild conditions. However, they do not show how to choose γ_j adaptively. In this paper, we adaptively choose γ_j by using the MM algorithm. The convergence of our algorithm can be proved following the idea in [13].

3 Optimization Algorithm

3.1 Details of the algorithm

We first solve the Group Lasso problem (14).

$$\hat{\boldsymbol{\beta}}^{GL}(\gamma) = \arg\min_{\boldsymbol{\beta}} R^{(GL)}(\boldsymbol{\beta}) = \arg\min_{\boldsymbol{\beta}} \left(\frac{1}{2N} \left\| \mathbf{y} - (\beta_0 + \sum_{j=1}^{J} \mathbf{X}_j \boldsymbol{\beta}_j) \right\|_2^2 + \gamma \sum_{j=1}^{J} \sqrt{p_j} \|\boldsymbol{\beta}_j\|_2 \right). \tag{14}$$

It serves as the basis to solve the problem (15).

$$\hat{\boldsymbol{\beta}}^{AGL}(\gamma) = \arg\min_{\boldsymbol{\beta}} R^{(AGL)}(\boldsymbol{\beta}) = \arg\min_{\boldsymbol{\beta}} \left(-\ell(\boldsymbol{\beta}) + \gamma \sum_{j=1}^{L} w_j \sqrt{p_j} \|\boldsymbol{\beta}_j\|_2 \right), \tag{15}$$

where $\ell(\boldsymbol{\beta})$ is the log-likelihood of logistic regression:

$$\ell(\boldsymbol{\beta}) = \frac{1}{N}\sum_{i=1}^{N}\left[y_i(\beta_0 + \sum_{j=1}^{L}\mathbf{X}_{ij}\boldsymbol{\beta}_j) - \log\left(1 + \exp(\beta_0 + \sum_{j=1}^{L}\mathbf{X}_{ij}\boldsymbol{\beta}_j)\right)\right]. \tag{16}$$

For convenience, we standardize the block matrix $\mathbf{X}_j$ such that $\frac{1}{N}\mathbf{X}_j^T\mathbf{X} = I_{p_j}$, where I_{p_j} is a $p_j \times p_j$ identity matrix. After model fitting, the estimated coefficients $\boldsymbol{\beta}$ will be transformed back in the original scale. Notice that the standardization can be done efficiently: The inner product of any two dummy variables within the same group is always zero. Thus, $\frac{1}{N}\mathbf{X}_j^T\mathbf{X}$ is diagonal. Simple scaling can convert it to an identity matrix. General orthogonalization techniques such as QR decomposition is not necessary.

Now let us consider a coordinate descent algorithm to solve (14). Suppose we have the estimation $\tilde{\beta}_0$ and $\tilde{\boldsymbol{\beta}}_l$ for $l \neq j$, and we like to optimize (14) with respect to $\boldsymbol{\beta}_j$. We compute the gradient at $\boldsymbol{\beta} = \tilde{\boldsymbol{\beta}}$ when $\tilde{\boldsymbol{\beta}}_j \neq \mathbf{0}$:

$$\frac{\partial R^{(GL)}}{\partial \boldsymbol{\beta}_j}|_{\boldsymbol{\beta}=\tilde{\boldsymbol{\beta}}} = -\frac{1}{N}\mathbf{X}_j^T(\mathbf{y} - \tilde{\beta}_0 - \sum_{l\neq j}\mathbf{X}_l\tilde{\boldsymbol{\beta}}_l - \mathbf{X}_j\boldsymbol{\beta}_j) + \gamma\sqrt{p_j}\frac{\boldsymbol{\beta}_j}{\|\boldsymbol{\beta}_j\|} = \mathbf{0}. \tag{17}$$

When $\tilde{\boldsymbol{\beta}}_j = \mathbf{0}$, we have

$$\| -\frac{1}{N}\mathbf{X}_j^T(\mathbf{y} - \tilde{\beta}_0 - \sum_{l\neq j}\mathbf{X}_l\tilde{\boldsymbol{\beta}}_l)\| \leq \gamma\sqrt{p_j}. \tag{18}$$

Expressions (17) and (18) are actually the Karush-Kuhn-Tucker conditions given in [10]. They can be rewritten as

$$\mathbf{r}_j = \boldsymbol{\beta}_j + \gamma\sqrt{p_j}\frac{\boldsymbol{\beta}_j}{\|\boldsymbol{\beta}_j\|}, \tag{19}$$

$$\|\mathbf{r}_j\| \leq \gamma\sqrt{p_j}, \tag{20}$$

where

$$\mathbf{r}_j = \frac{1}{N}\mathbf{X}_j^T(\mathbf{y} - \tilde{\mathbf{y}}^{(j)}), \tag{21}$$

$$\tilde{\mathbf{y}}^{(j)} = \tilde{\beta}_0 + \sum_{l\neq j}\mathbf{X}_l\tilde{\boldsymbol{\beta}}_l. \tag{22}$$

After combining (19) and (20), the coordinate-wise update has the form

$$\boldsymbol{\beta}_j = \left(1 - \frac{\gamma\sqrt{p_j}}{\|\mathbf{r}_j\|}\right)_+ \mathbf{r}_j, \tag{23}$$

where $(\cdot)_+$ is the operator

$$(v)_+ = \begin{cases} v & \text{if } v \geq 0 \\ 0 & \text{if } v < 0 \end{cases}. \tag{24}$$

Therefore, iteratively updating $\boldsymbol{\beta}_j$ by (23) for $j = 1, \cdots, L$ gives the solution of (14).

Now let us consider Logistic Group Lasso problem (15). We form a quadratic approximation to the log-likelihood based on current estimation $\tilde{\boldsymbol{\beta}}$:

$$\ell(\boldsymbol{\beta}) \approx \ell_Q(\boldsymbol{\beta}) = \ell(\tilde{\boldsymbol{\beta}}) + (\boldsymbol{\beta} - \tilde{\boldsymbol{\beta}})^T \nabla \ell(\tilde{\boldsymbol{\beta}}) + \frac{1}{2}(\boldsymbol{\beta} - \tilde{\boldsymbol{\beta}})^T \mathbf{H}(\boldsymbol{\beta} - \tilde{\boldsymbol{\beta}}). \quad (25)$$

Here $\nabla \ell(\tilde{\boldsymbol{\beta}})$ and $\mathbf{H}$ are the gradient and the Hessian matrix of $\ell(\cdot)$ evaluated at $\tilde{\boldsymbol{\beta}}$, respectively:

$$\nabla \ell(\tilde{\boldsymbol{\beta}}) = \frac{1}{N}\mathbf{X}^T(\mathbf{y} - \tilde{\mathbf{p}}), \quad (26)$$

$$\mathbf{H} = \frac{1}{N}\mathbf{X}^T\mathbf{U}\mathbf{X}, \quad (27)$$

where $\mathbf{U}$ is a diagonal matrix with diagonal element $u_i = \tilde{p}(\mathbf{X}_i)(1 - \tilde{p}(\mathbf{X}_i))$ and

$$\tilde{p}(\mathbf{X}_i) = \frac{1}{1 + \exp(-\tilde{\beta}_0 - \sum_j \mathbf{X}_{ij}\tilde{\beta}_j)}. \quad (28)$$

Rearranging expressions (25) $\sim$ (28), we obtain

$$\ell_Q(\boldsymbol{\beta}) = -\frac{1}{2N}\sum_i^N u_i(z_i - \beta_0 - \sum_j^L \mathbf{X}_{ij}\boldsymbol{\beta}_j)^2 + C(\tilde{\boldsymbol{\beta}}), \quad (29)$$

where

$$z_i = \tilde{\beta}_0 + \sum_j \mathbf{X}_j\tilde{\boldsymbol{\beta}}_j + \frac{y - \tilde{p}(\mathbf{X}_i)}{\tilde{p}(\mathbf{X}_i)(1 - \tilde{p}(\mathbf{X}_i))}, \quad (30)$$

$$u_i = \tilde{p}(\mathbf{X}_i)(1 - \tilde{p}(\mathbf{X}_i)), \quad (31)$$

$$\tilde{p}(\mathbf{X}_i) = \frac{1}{1 + \exp\left(-\tilde{\beta}_0 - \sum_j \mathbf{X}_j\tilde{\boldsymbol{\beta}}_j\right)}, \quad (32)$$

and $C(\tilde{\boldsymbol{\beta}})$ is a constant term only involving $\tilde{\boldsymbol{\beta}}$. Then, the problem (15) is converted to the least square problem with a Group Lasso constraint:

$$\min_{\boldsymbol{\beta}} \{-\ell_Q(\beta_0, \boldsymbol{\beta}) + \gamma \sum_{j=1}^L w_j\sqrt{p_j}\|\boldsymbol{\beta}_j\|_2\}. \quad (33)$$

Further making use of the upper bound of Hessian terms u_i as given in [3], we set $u_i = 0.25$ in (29) which saves computation effort for calculating the Hessian matrix, then problem (33) becomes

$$\min_{\boldsymbol{\beta}} \{-\frac{1}{2N}\sum_i^N 0.25(z_i - \beta_0 - \sum_j^L \mathbf{X}_{ij}\boldsymbol{\beta}_j)^2 + \gamma\sum_{j=1}^L w_j\sqrt{p_j}\|\boldsymbol{\beta}_j\|_2\}. \quad (34)$$

Problem (34) is exactly in the form (14) with equal weight, and thus can be solved by coordinate descent updating formula

$$\boldsymbol{\beta}_j = \left(1 - \frac{4\gamma w_j\sqrt{p_j}}{\|\mathbf{r}_j\|}\right)_+ \mathbf{r}_j, \quad (35)$$

where $\mathbf{r}_j$ is the vector with the i-th elements $\frac{1}{N}\mathbf{X}_{ij}^T\left(z_i - \tilde{\beta}_0 - \sum_{l\neq j}\mathbf{X}_{il}\tilde{\boldsymbol{\beta}}_l\right)$. The intercept β_0 is not penalized. It is initialized as $\tilde{\beta}_0 = \log(\frac{p_0}{1-p_0})$, where $p_0 = \sum_i y_i/N$. We update it by using $\beta_0 \leftarrow \tilde{\beta}_0 + \frac{\sum_i(y_i - \tilde{p}_i)}{\sum_i \tilde{p}_i(1-\tilde{p}_i)}$.

3.2 Implement issues

The parameter γ is typically unknown. Here we use cross-validation to determine its value. As cross-validation needs a sequence of γ values, we construct the sequence as follows: We set the maximum value and the minimum value as $\gamma_{max} = \max_j \frac{\|\mathbf{X}_j^T(\mathbf{y}-p_0)\|_2}{Nw_j\sqrt{p_j}}$, $\gamma_{min} = \epsilon\gamma_{max}$, respectively. Then, we construct a sequence of γ values decreasing from γ_{max} to γ_{min} uniformly on log scale $\boldsymbol{\gamma} = [\gamma_{max} = \gamma_1 > \gamma_2 > \cdots > \gamma_K = \gamma_{min}]$. To solve $\boldsymbol{\beta}(\gamma_k)$ $k = 1, \ldots, K$, we exploit the *warm starts* technique: by using $\boldsymbol{\beta}(\gamma_k)$ as the initial point for $\boldsymbol{\beta}(\gamma_{k+1})$. In doing so, we are able to do cross-validation on γ values efficiently. In our experiment, we typically set $K = 100$, $\epsilon = 0.001$ when $N > L$, when $N < L$, we set $\epsilon = 0.05$.

We also know that many $\boldsymbol{\beta}_j$s remain as zero during the whole optimization process from expression (35). Naive cycling optimization on all groups in iteration process loses efficiency. We make use of this property as in [2], which leads to considerable speedup:

- Step 1: We complete a cycle through all groups, and identify the active set (nonzero $\boldsymbol{\beta}_j$).
- Step 2: We iterative only on the active set until convergence.
- Step 3: We form a cycling through all groups again. If this does not change the active set, the optimization process converges; otherwise go to Step 2.

4 More Results

4.1 Comparison with MDR

We choose MDR [6] for comparison because MDR is a very popular method. Our approach is similar to MDR in the sense that both methods enumerate all possible interactions during model fitting process. The difference is that our approach analyzes all interactions simultaneously, while MDR searches the best model in a sequential manner.

During the comparison with MDR, we use six benchmark epitasis models [5] (details are in the supplementary document). Various sources of noise, such as genotyping error (GE), missing data (MS), phenocopy (PC), and genetic heterogeneity (GH) are added in the simulated data. The reader is referred to [5] for details of these six models and data simulation procedure.

The MDR algorithm is one of the most popular tools for gene-gene interaction detection. It has a very good power for identifying associated SNPs in the presence of various kinds of noise except that its power decreases significantly in the presence of genetic heterogeneity. Our method is similar to MDR in the sense that both methods enumerate all possible interactions. The difference is that MDR searches all interactions in a sequential manner and pick the best one, while our method simultaneously analyzes all interactions. The comparison results are shown in Table 1. The novelty of our method is that it significantly increases the power when the genetic heterogeneity is present. Concretely, for Model 2-1, Model 2-3, Model 2-4, Model

2-5 and Model 2-6, the power of MDR drops from greater than 80% to only 5%. For Model 2-2, its power drops from 100% to 41%. While the power of our method drops from around 100% to 50% for Model 2-3, Model 2-4, Model 2-5 and Model 2-6, and the power remains the same for Model 2-1 and Model 2-2. This clearly shows that our method is more robust in the presence of genetic heterogeneities.

MDR searches for all possible combinations of SNPs. Thus, it is able to detect high-order interactions even when the main effects and low-order effects are not significant. However, it is difficult for MDR to characterize additive structures between associated SNPs. Genetic heterogeneity is such a case that MDR performs poorly.

In contrast, our method does better when genetic heterogeneities are present. The reason is that our model is able to approximate genetic heterogeneity model by making use of the additive structure. Given the genetic heterogeneity model:

$$\log \frac{Pr(y=0|SNP_{k_1}, SNP_{k_2})}{Pr(y=1|SNP_{k_1}, SNP_{k_2})} = \sum_{g \in G_2} \alpha^g_{k_1,k_2} \cdot I\left((SNP_{k_1}, SNP_{k_2}) = g\right), \tag{36}$$

$$\log \frac{Pr(y=0|SNP_{k_3}, SNP_{k_4})}{Pr(y=1|SNP_{k_3}, SNP_{k_4})} = \sum_{g \in G_2} \alpha^g_{k_3,k_4} \cdot I\left((SNP_{k_3}, SNP_{k_4}) = g\right), \tag{37}$$

where α^g depends on a particular disease model and $I(\text{expression})$ is the indicator function:

$$I(\text{expression}) = \begin{cases} 1 & \text{if expression is true,} \\ 0 & \text{otherwise.} \end{cases}$$

Suppose half of the disease samples comes from model (36) and another half comes from the model (37). Our model approximates the genetic heterogeneity model in the following form:

$$\begin{aligned} &\log \frac{Pr(y=0|SNP_{k_1}, SNP_{k_2}, SNP_{k_3}, SNP_{k_4})}{Pr(y=1|SNP_{k_1}, SNP_{k_2}, SNP_{k_3}, SNP_{k_4})} \\ &= \sum_{g \in G_2} \left[\beta^g_{k_1,k_2} \cdot I\left((SNP_{k_1}, SNP_{k_2}) = g\right) + \beta^g_{k_3,k_4} \cdot I\left((SNP_{k_3}, SNP_{k_4}) = g\right)\right], \end{aligned} \tag{38}$$

where the coefficients $\beta^g_{k_1,k_2}$ and $\beta^g_{k_3,k_4}$ can be estimated by our proposed method. Noise SNPs are unlikely to enter the final model due to the sparsity penalty.

We show a typical coefficient pattern in Fig. 1 when genetic heterogeneity is present. The interaction term (SNP_{k_1}, SNP_{k_2}) enters the model first and is followed by (SNP_{k_3}, SNP_{k_4}). The interaction terms borrow strength from each other and enter the model. Other noise terms enter the model gradually as γ decreases. Finally, the signals of (SNP_{k_1}, SNP_{k_2}) and (SNP_{k_3}, SNP_{k_4}) become unobvious among noise terms. We also show the coefficients estimated at γ_* which is determined by cross-validation. One can see that the signals of (SNP_{k_1}, SNP_{k_2}) and (SNP_{k_3}, SNP_{k_4}) are much stronger than noise terms (the middle panel of Fig. 1). Reweighted estimation weakens noise terms further (the right panel of Fig. 1).

Table 1: Power comparison between MDR and our method in detecting the interacting SNPs. Various sources of noise are simulated (GE, 5% genotyping error; GH, 50% genetic heterogeneity; PC, 50% phenocopy; MS, 5% missing data). Notice that the results involving the GH noise are marked by gray color.

**MDR/Our method**	**Power(%)**					
Source of noise	Model 2-1	Model 2-2	Model 2-3	Model 2-4	Model 2-5	Model 2-6
None	100/100	100/100	99/100	99/100	82/99	84/100
GE	100/100	100/100	100/100	97/100	80/96	92/100
GH	3/100	41/100	2/53	3/56	4/46	4/60
PC	90/97	99/100	45/50	32/38	30/46	32/62
MS	100/100	100/100	99/100	97/100	82/97	87/100
GE+GH	4/99	41/100	2/53	3/56	4/46	6/60
GE+PC	94/96	99/99	41/51	48/50	28/57	33/64
GE+MS	100/100	100/100	98/100	98/100	74/96	84/100
GH+PC	0/30	1/59	0/13	0/5	0/24	0/14
GH+MS	5/99	38/100	0/56	2/53	4/43	6/59
PC+MS	96/97	99/100	42/50	43/52	14/35	16/48
GE+GH+PC	1/36	1/58	0/10	0/8	0/13	0/16
GE+GH+MS	6/98	34/100	2/49	1/51	3/82	7/52
GH+PC+MS	0/31	0/61	0/10	0/6	0/14	0/19
GE+PC+MS	94/97	100/100	48/48	42/47	18/38	16/46
GE+GH+PC+MS	0/33	1/54	0/12	1/14	0/12	0/16

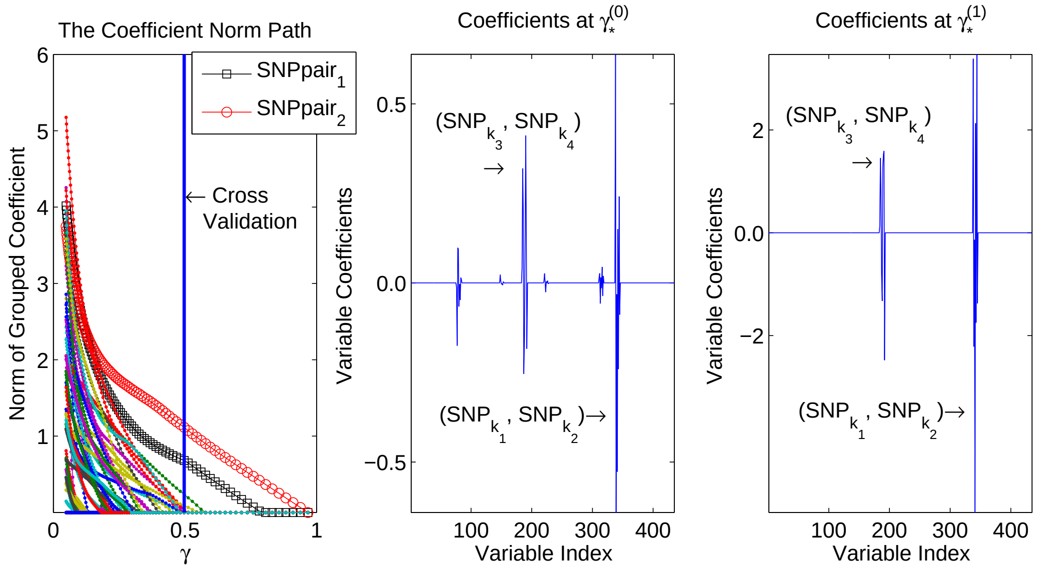

Figure 1: The path of coefficient norms and the coefficients evaluated at $\gamma_*^{(m)}$. We use cross validation to determine the value of $\gamma_*^{(m)}$.

4.2 Comparison with BEAM

4.2.1 The influence of main effects

In the paper, we show that BEAM performs poorly when main effects are absent. Here we conduct simulation study showing that the performance of BEAM depends on the main effects. Our simulation is based on the XOR model given in Table 2 with $\alpha = 1$ and $\theta = 2$. The minor allele frequency (MAF) varies from 0.1 to 0.5 such that the main effect of the XOR model decreases until no main effect is present, as shown in Fig. 2. We simulate 100 datasets. Each dataset contains 400 samples and 1000 SNPs. The results of BEAM and AGL are shown in Fig. 3:

- These two methods have comparable power for detecting main effects.
- BEAM has a low power to detect interactions when MAF$= 0.3, 0.4, 0.5$. The reason is that the two associated SNPs with weak main effects are difficult to be sampled. When MAF$= 0.2$, the two associated SNPs with noticeable main effects are more likely to be detected. Thus, the power of BEAM increases dramatically. When MAF$= 0.1$, it is easier for BEAM to detect the two associated SNPs but BEAM prefers to recognize them as main effects rather than interactions.
- The performance of AGL is robust for different MAFs.

	AA	Aa	aa
BB	α	$\alpha(1+\theta)$	α
Bb	$\alpha(1+\theta)$	α	$\alpha(1+\theta)$
bb	α	$\alpha(1+\theta)$	α

Table 2: The XOR model: $\alpha = 1$ and $\theta = 2$ is used in the simulation study. The minor allele frequency (MAF) varies from 0.1 to 0.5 such that the main effect of the XOR model decreases until no main effect is present.

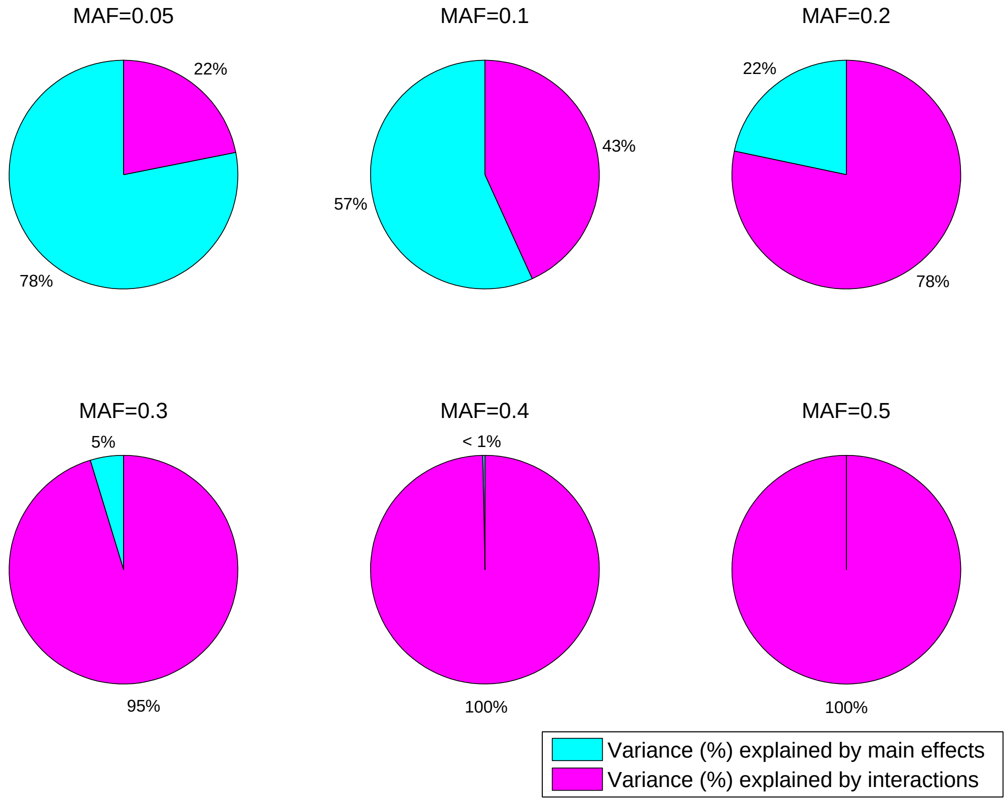

Figure 2: The percentage variance of XOR model (Table 2) explained by main effects and interactions with different MAFs. As the MAF increases from 0.05 to 0.5, the main effect decreases until zero.

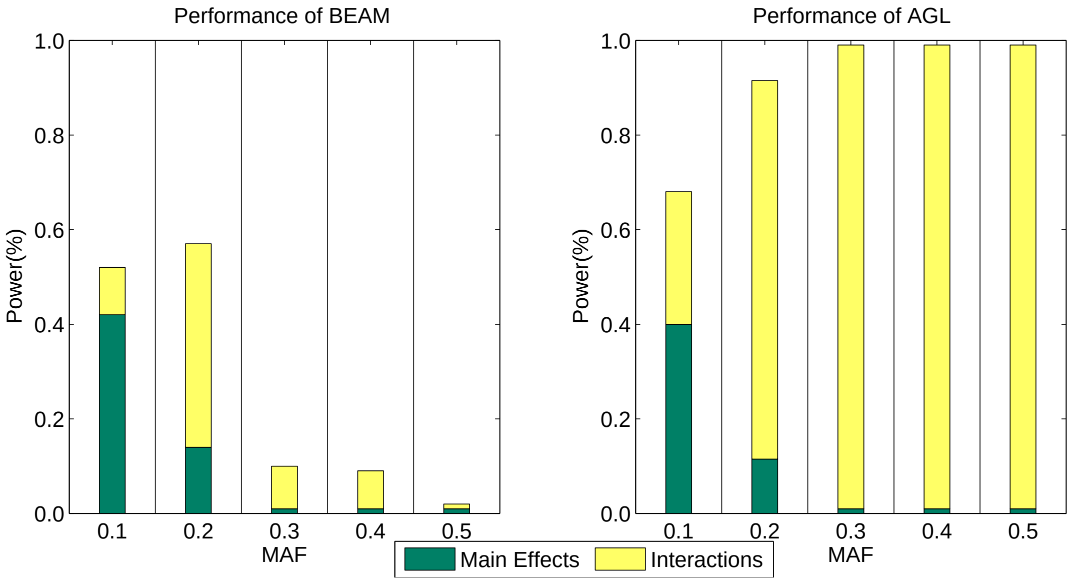

Figure 3: Comparison results of BEAM and Adaptive Group Lasso (AGL) on the XOR model for MAF$= 0.1$, $= 0.2$, $= 0.3$, $= 0.4$ and $= 0.5$. As the MAF increases from 0.1 to 0.5, the main effect decreases until zero. BEAM has a low power to detect interactions when MAF $= 0.3$, 0.4, and 0.5. The power of BEAM increases dramatically when MAF $= 0.2$. When MAF $=$ 0.1, it is easier for BEAM to detect the two associated SNPs but BEAM prefers to recognize them as main effects rather than interactions.

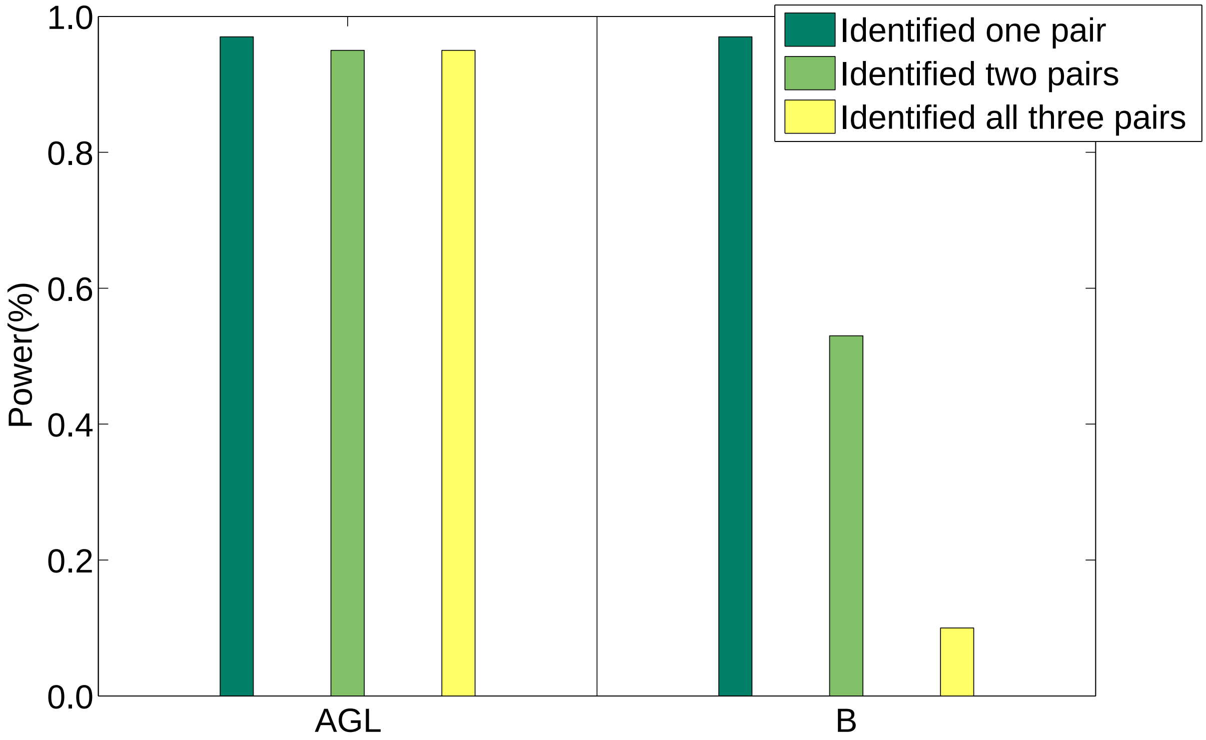

Figure 4: Comparison between our method and BEAM based on the XOR model (Table 2). BEAM (B) loses its power when detecting multiple interaction pairs, while our AGL keeps its power.

4.2.2 Detecting multiple interactions

In order to have a fair comparison regarding the power of detecting multiple interactions, we simulate interacting SNPs with noticeable main effects since BEAM has a low power when the main effect is weak. We design our experiment based on the XOR model shown in Table 2. Fig. 2 shows that the main effect of the XOR model tends to be zero as the MAF increases from 0.1 to 0.5. We choose MAF to be 0.2 such that interacting SNPs have noticeable main effects. Under this setting, it is easier for BEAM to sample those associated SNPs. We simulate 1000 samples and 500 SNPs. Six of 500 SNPs form three groups of interacting SNPs. Each group has the same characteristics as described by the XOR model. Fig. 4 shows the result. BEAM loses its power when detecting more than one interacting groups. Concretely, the power of BEAM is about 97% when identifying one interactions. But the power quickly drops to around 50% when identifying two interactions and further drops to 10% for identifying all three interactions. Our method keeps the high power when identifying all three interactions.

SNPs	Location	Related Genes	P-value
rs7539166	1p36	TMEM51	$< 10^{-30}$
rs384843	1p36	NBPF3	5.2×10^{-9}
rs7516721	1p36	GPR3	7.7×10^{-14}
rs3737819	1p34	COL9A2	2.8×10^{-9}
rs17107203	1p32	unknown	2.2×10^{-13}
rs1920164	1p31	unknown	2.2×10^{-9}
rs12118611	1q24	NME7	$< 10^{-30}$
rs4658037	1q31	KCNT2	$< 10^{-30}$
rs1999987	1q42	DISP1	5.8×10^{-19}
rs5744138	1q41	TLR5	4.2×10^{-8}
rs1640803	1q41	unknown	2.8×10^{-9}
rs851148	1q41	unknown	6.5×10^{-11}
rs1772272	1q41	unknown	1.7×10^{-10}

Table 3: Identified SNPs on Chromosome 1.

4.3 WTCCC RA data results

We provide the result of WTCCC Rheumatoid Arthritis (RA) data set. These results are analyzed under main effect model in the chromosome-wise manner. These results are given in Table 3 $\sim$ Table 10.

5 Models Used in Simulation Studies

5.1 Models used for comparison with MDR

We provide models used for comparison with MDR in Table 11.

5.2 Models used in comparison with BEAM

The epistatic models used in Section 3.1.3 are given in Table 12, Table 13, Table 14 and Table 15. All of these models can be found in [8].

SNPs	Location	Related Genes	P-value
rs1572075	4p16	ZNF718	$< 10^{-30}$
rs7694697	4p16	SORCS2	$< 10^{-30}$
rs4330351	4p16	SORCS2	1.0×10^{-10}
rs1444360	4p16	AC116049	$< 10^{-30}$
rs16861011	4p12	TXK	6.2×10^{-9}
rs7699492	4q23	TSPAN5	$< 10^{-30}$
rs17027886	4q23	TSPAN5	$< 10^{-30}$
rs17050351	4q32	AC092643	$< 10^{-30}$
rs13104196	4q32	PDGFC	$< 10^{-30}$
rs17525479	4q32	PDGFC	$< 10^{-30}$

Table 4: Identified SNPs on Chromosome 4.

SNPs	Location	Related Genes	P-value
rs9296921	6p23	CD83	$< 10^{-30}$
rs4959053	6p21	PSORS1C1	$< 10^{-30}$
rs6457617	6p21	MHC region	1.3×10^{-15}
rs9387380	6q21	FRK	3.4×10^{-15}
rs17165379	6q25	ZDHHC14	$< 10^{-30}$

Table 5: Identified SNPs on Chromosome 6.

SNPs	Location	Related Genes	P-value
rs1494192	7p15	NPY	$< 10^{-30}$
rs17356657	7q31	C7orf58	2.2×10^{-16}
rs12538802	7q31	CADPS2	4.7×10^{-13}
rs6973565	7q32	PLXNA4	1.2×10^{-9}
rs10250029	7q32	CHCHD3	$< 10^{-30}$
rs7789415	7q36	PTPRN2	3.6×10^{-12}

Table 6: Identified SNPs on Chromosome 7.

SNPs	Location	Related Genes	P-value
rs10751815	Chr10p15	ADARB2	$< 10^{-30}$
rs4266996	Chr10p15	unknown	$< 10^{-30}$
rs17147777	Chr10p15	unknown	$< 10^{-30}$
rs4750402	Chr10p14	FRMD4A	1.2×10^{-13}
rs2121526	10q11	PCDH15	$< 10^{-30}$
rs16925310	10q21	PBLD	4.6×10^{-10}
rs11185776	10q23	PANK1	4.7×10^{-9}

Table 7: Identified SNPs on Chromosome 10.

SNPs	Location	Related Genes	P-value
rs2880301	13q12	TPTE2	1.0×10^{-10}
rs4379926	13q12	unknown	$< 10^{-30}$
rs17086772	13q12	LOC341784	$< 10^{-30}$

Table 8: Identified SNPs on Chromosome 13.

SNPs	Location	Related Genes	P-value
rs4111253	21p11	BAGE	$< 10^{-30}$
rs8129909	21q22	IGSF5	$< 10^{-30}$
rs16999716	21q22	DSCAM	9.2×10^{-9}
rs4542939	21q22	DSCAM	8.3×10^{-9}
rs13047947	21q22	PDE9A	$< 10^{-30}$

Table 9: Identified SNPs on Chromosome 21.

SNPs	Location	Related Genes	P-value
rs140344	22q12	unknown	$< 10^{-30}$
rs5749509	22q12	SYN3	$< 10^{-30}$
rs6518796	22q12	SYN3	$< 10^{-30}$

Table 10: Identified SNPs on Chromosome 22.

Table 11: Two-locus penetrance functions of six two-locus models that exhibit epistatic interactions in the absence of main effects [5]. Here p and q denote allele frequencies of A and B, respectively.

Model 2-1	$p = 0.5, q = 0.5$		
	AA	Aa	aa
BB	0	0.1	0
Bb	0.1	0	0.1
bb	0	0.1	0

Model 2-2	$p = 0.5, q = 0.5$		
	AA	Aa	aa
BB	0	0	0.1
Bb	0	0.05	0
bb	0.1	0	0

Model 2-3	$p = 0.25, q = 0.75$		
	AA	Aa	aa
BB	0.08	0.07	0.05
Bb	0.1	0	0.1
bb	0.03	0.1	0.04

Model 2-4	$p = 0.25, q = 0.75$		
	AA	Aa	aa
BB	0	0.01	0.09
Bb	0.04	0.01	0.08
bb	0.07	0.09	0.03

Model 2-5	$p = 0.1, q = 0.9$		
	AA	Aa	aa
BB	0.07	0.05	0.02
Bb	0.05	0.09	0.01
bb	0.02	0.01	0.03

Model 2-6	$p = 0.1, q = 0.9$		
	AA	Aa	aa
BB	0.09	0.001	0.02
Bb	0.08	0.07	0.005
bb	0.003	0.007	0.02

$h^2 = 0.3, MAF = 0.2$				$h^2 = 0.3, MAF = 0.4$			
Model epi1	AA	Aa	aa	Model epi6	AA	Aa	aa
BB	0.500	0.926	0.615	BB	0.891	0.362	0.480
Bb	0.895	0.131	0.647	Bb	0.213	0.829	0.601
bb	0.858	0.160	0.999	bb	0.925	0.267	0.685
$h^2 = 0.3, MAF = 0.2$				$h^2 = 0.3, MAF = 0.4$			
Model epi2	AA	Aa	aa	Model epi7	AA	Aa	aa
BB	0.413	0.851	0.535	BB	0.077	0.689	0.417
Bb	0.831	0.008	0.580	Bb	0.763	0.150	0.491
bb	0.692	0.268	0.736	bb	0.196	0.657	0.247
$h^2 = 0.3, MAF = 0.2$				$h^2 = 0.3, MAF = 0.4$			
Model epi3	AA	Aa	aa	Model epi8	AA	Aa	aa
BB	0.455	0.848	0.897	BB	0.132	0.793	0.274
Bb	0.890	0.088	0.016	Bb	0.799	0.213	0.514
bb	0.562	0.686	0.467	bb	0.255	0.528	0.793
$h^2 = 0.3, MAF = 0.2$				$h^2 = 0.3, MAF = 0.4$			
Model epi4	AA	Aa	aa	Model epi9	AA	Aa	aa
BB	0.609	0.980	0.980	BB	0.611	0.104	0.759
Bb	0.993	0.300	0.275	Bb	0.180	0.674	0.019
bb	0.876	0.483	0.683	bb	0.532	0.189	0.681
$h^2 = 0.3, MAF = 0.2$				$h^2 = 0.3, MAF = 0.4$			
Model epi5	AA	Aa	aa	Model epi10	AA	Aa	aa
BB	0.486	0.963	0.512	BB	0.091	0.827	0.863
Bb	0.941	0.006	0.899	Bb	0.869	0.393	0.415
bb	0.691	0.541	0.614	bb	0.738	0.508	0.363

Table 12: Epistatic models with $h^2 = 0.3, MAF = 0.2, 0.4$.

$h^2 = 0.2, MAF = 0.2$				$h^2 = 0.2, MAF = 0.4$			
Model epi11	AA	Aa	aa	Model epi16	AA	Aa	aa
BB	0.428	0.757	0.812	BB	0.356	0.891	0.809
Bb	0.788	0.132	0.044	Bb	0.955	0.508	0.611
bb	0.559	0.548	0.373	bb	0.617	0.755	0.630
$h^2 = 0.2, MAF = 0.2$				$h^2 = 0.2, MAF = 0.4$			
Model epi12	AA	Aa	aa	Model epi17	AA	Aa	aa
BB	0.507	0.842	0.605	BB	0.086	0.536	0.641
Bb	0.845	0.162	0.629	Bb	0.677	0.275	0.096
bb	0.581	0.678	0.729	bb	0.219	0.413	0.712
$h^2 = 0.2, MAF = 0.2$				$h^2 = 0.2, MAF = 0.4$			
Model epi13	AA	Aa	aa	Model epi18	AA	Aa	aa
BB	0.577	0.247	0.428	BB	0.855	0.339	0.772
Bb	0.227	0.928	0.578	Bb	0.513	0.651	0.607
bb	0.586	0.262	0.158	bb	0.250	0.999	0.154
$h^2 = 0.2, MAF = 0.2$				$h^2 = 0.2, MAF = 0.4$			
Model epi14	AA	Aa	aa	Model epi19	AA	Aa	aa
BB	0.340	0.637	0.654	BB	0.506	0.838	0.024
Bb	0.689	0.017	0.041	Bb	0.603	0.454	0.957
bb	0.242	0.866	0.403	bb	0.729	0.427	0.753
$h^2 = 0.2, MAF = 0.2$				$h^2 = 0.2, MAF = 0.4$			
Model epi15	AA	Aa	aa	Model epi20	AA	Aa	aa
BB	0.387	0.726	0.734	BB	0.393	0.764	0.664
Bb	0.749	0.090	0.034	Bb	0.850	0.398	0.733
bb	0.551	0.401	0.724	bb	0.406	0.927	0.147

Table 13: Epistatic models with $h^2 = 0.2, MAF = 0.2, 0.4$.

$h^2 = 0.1, MAF = 0.2$				$h^2 = 0.1, MAF = 0.4$			
Model epi21	AA	Aa	aa	Model epi26	AA	Aa	aa
BB	0.463	0.703	0.431	BB	0.137	0.484	0.187
Bb	0.653	0.277	0.806	Bb	0.482	0.166	0.365
bb	0.830	0.008	0.129	bb	0.193	0.361	0.430

$h^2 = 0.1, MAF = 0.2$				$h^2 = 0.1, MAF = 0.4$			
Model epi22	AA	Aa	aa	Model epi27	AA	Aa	aa
BB	0.319	0.507	0.569	BB	0.469	0.198	0.754
Bb	0.553	0.105	0.045	Bb	0.337	0.502	0.141
bb	0.203	0.777	0.280	bb	0.339	0.453	0.285

$h^2 = 0.1, MAF = 0.2$				$h^2 = 0.1, MAF = 0.4$			
Model epi23	AA	Aa	aa	Model epi28	AA	Aa	aa
BB	0.627	0.393	0.335	BB	0.478	0.311	0.864
Bb	0.396	0.779	0.953	Bb	0.387	0.579	0.263
bb	0.174	0.842	0.106	bb	0.634	0.436	0.138

$h^2 = 0.1, MAF = 0.2$				$h^2 = 0.1, MAF = 0.4$			
Model epi24	AA	Aa	aa	Model epi29	AA	Aa	aa
BB	0.297	0.540	0.441	BB	0.068	0.299	0.017
Bb	0.541	0.072	0.278	Bb	0.289	0.044	0.285
bb	0.434	0.293	0.228	bb	0.048	0.262	0.174

$h^2 = 0.1, MAF = 0.2$				$h^2 = 0.1, MAF = 0.4$			
Model epi25	AA	Aa	aa	Model epi30	AA	Aa	aa
BB	0.332	0.562	0.573	BB	0.539	0.120	0.258
Bb	0.583	0.112	0.147	Bb	0.165	0.378	0.325
bb	0.399	0.496	0.033	bb	0.123	0.426	0.276

Table 14: Epistatic models with $h^2 = 0.1, MAF = 0.2, 0.4$.

$h^2 = 0.05, MAF = 0.2$				$h^2 = 0.05, MAF = 0.4$			
Model epi31	AA	Aa	aa	Model epi36	AA	Aa	aa
BB	0.492	0.664	0.481	BB	0.002	0.155	0.214
Bb	0.642	0.330	0.746	Bb	0.199	0.071	0.022
bb	0.656	0.396	0.000	bb	0.081	0.122	0.135

$h^2 = 0.05, MAF = 0.2$				$h^2 = 0.05, MAF = 0.4$			
Model epi32	AA	Aa	aa	Model epi37	AA	Aa	aa
BB	0.499	0.639	0.765	BB	0.188	0.020	0.171
Bb	0.666	0.389	0.083	Bb	0.032	0.174	0.059
bb	0.543	0.527	0.953	bb	0.134	0.087	0.092

$h^2 = 0.05, MAF = 0.2$				$h^2 = 0.05, MAF = 0.4$			
Model epi33	AA	Aa	aa	Model epi38	AA	Aa	aa
BB	0.212	0.350	0.116	BB	0.005	0.179	0.251
Bb	0.336	0.054	0.495	Bb	0.211	0.100	0.026
bb	0.227	0.273	0.495	bb	0.156	0.098	0.156

$h^2 = 0.05, MAF = 0.2$				$h^2 = 0.05, MAF = 0.4$			
Model epi34	AA	Aa	aa	Model epi39	AA	Aa	aa
BB	0.805	0.683	0.638	BB	0.174	0.321	0.154
Bb	0.657	0.936	0.989	Bb	0.223	0.254	0.245
bb	0.850	0.564	0.866	bb	0.448	0.025	0.424

$h^2 = 0.05, MAF = 0.2$				$h^2 = 0.05, MAF = 0.4$			
Model epi35	AA	Aa	aa	Model epi40	AA	Aa	aa
BB	0.638	0.488	0.383	BB	0.098	0.219	0.302
Bb	0.464	0.765	0.957	Bb	0.302	0.126	0.121
bb	0.580	0.562	0.719	bb	0.053	0.308	0.136

Table 15: Epistatic models with $h^2 = 0.05, MAF = 0.2, 0.4$.

References

[1] J. Fan and R. Li. Variable selection via nonconcave penalized likelihood and its oracle properties. *Journal of the American Statistical Association.*, 96:1348–1360, 2001.

[2] J. Friedman, T. Hastie, and R. Tibshirani. Regularization paths for generalized linear models via coordinate descent. *Technical Report, Stanford University*, 2008.

[3] B. Krishnapuram and A. J. Hartemink. Sparse multinomial logistic regression: Fast algorithms and generalization bounds. *IEEE Trans. Pattern Analsis and Machine Intellegence*, 2005.

[4] K. Lange, D. Hunter, and I. Yang. Optimization transfer using surrogate objective functions. *Journal of Computational and Graphical Statistics*, 9:1–59, 2000.

[5] M. D. Ritchie, L. W. Hahn, and J. H. Moore. Power of multifactor dimensionality reduction for detecting gene-gene interactions in the presence of genotyping error, missing data, phenocopy, and genetic heterogeneity. *Genetic Epidemiology*, 24:150–157, 2003.

[6] M.D. Ritchie, L.W. Hahn, N. Roodi, L.R Bailey, W.D. Dupont, F.F. Parl, and J.H. Moore. Multifactor-dimensionality reduction reveals high-order interactions among estrogenmetabolism genes in sporadic breast cancer. *The American Journal of Human Genetics*, 69:138–147, 2001.

[7] R. Tibshirani. Regression shrinkage and selection via the Lasso. *Journal of the Royal Statistical Society, series B*, 58:267–288, 1996.

[8] D.R. Velez, B.C. White, A.A. Motsinger, W.S. Bush, M.D. Ritchie, S.M. Williams, and J.H. Moore. A balanced accuracy function for epistasis modeling in imbalanced datasets using multifactor dimensionality reduction. *Genetic Epidemiology*, 31:306–315, 2007.

[9] H. Wang and C. Leng. A note on adaptive group lasso. *Computational Statistics and Data Analysis*, 52:5277–5286, 2008.

[10] M. Yuan and Y. Lin. Model selection and estimation in regression with grouped variables. *Journal of the Royal Statistical Society: Series B*, 68(1):49–67, 2006.

[11] H. Zou. The adaptive lasso and its oracle properties. *Journal of the American Statistical Association.*, 101:1418–1429, 2006.

[12] H. Zou and T. Hastie. Regularization and variable selection via the elastic net. *Journal of the Royal Statistical Society: Series B*, 67:301–320, 2005.

[13] H. Zou and R. Li. One-step sparse estimates in nonconcave penalized likelihood models. *The Annals of Statistics*, 36(4):1509–1533, 2008.
